# Assessing grey matter structural alterations in systemic lupus erythematosus using synthetic MRI

**DOI:** 10.1136/lupus-2025-001505

**Published:** 2025-07-13

**Authors:** Kemei Deng, Chengli Wu, Yuhong Qin, Wei Cui, Jing Wen, Muliang Jiang, Liling Long, Bihong T Chen

**Affiliations:** 1 Department of Radiology, The First Affiliated Hospital of Guangxi Medical University, Nanning, Guangxi, China; 2 GE HealthCare China, Beijing, China; 3 Department of Rheumatology and Immunology, The First Affiliated Hospital of Guangxi Medical University, Nanning, Guangxi, China; 4 City of Hope National Medical Center, Duarte, California, USA

**Keywords:** Systemic Lupus Erythematosus, Magnetic Resonance Imaging, Autoimmune Diseases

## Abstract

**ABSTRACT:**

**Objectives:**

To assess brain grey matter alterations in patients with SLE and their correlation with neuropsychological testing using synthetic MRI (SyMRI).

**Methods:**

This prospective study enrolled patients with SLE and age, gender and education-matched healthy controls (HC). Study assessments included brain MRI using SyMRI and neuropsychological tests: Mini-Mental State Examination (MMSE), Montreal Cognitive Assessment (MoCA), Digit Span Test, Self-Rating Anxiety Scale and Self-Rating Depression Scale (SDS). SyMRI post-processing and Automated Anatomical Labeling were used for grey matter mapping. Correlation analysis was performed to assess the relationship between brain grey matter structural alterations and neuropsychological testing.

**Results:**

77 patients with SLE (57 non-neuropsychiatric SLE (non-NPSLE), 20 NPSLE) and 29 HC participants were enrolled. Patients with SLE showed reduced grey matter volume compared with HC (p<0.05). The NPSLE group exhibited more extensive increases in longitudinal (T1) and transverse (T2) relaxation times in grey matter than the non-NPSLE group (p<0.001). Proton density values were lower in patients with SLE (p<0.001). Lower brain parenchymal volume correlated with higher SLE Disease Activity Index (p<0.05). Lower MMSE/MoCA scores correlated with increased T1/T2 in the left medial cingulate and paracingulate gyri (p<0.05). Higher SDS scores correlated with increased T1/T2 in the left calcarine fissure and surrounding cortex (p<0.05). These changes were also linked to disease markers (C3, C4, immunoglobulin M, erythrocyte sedimentation rate) (p<0.05).

**Conclusions:**

Grey matter alterations in patients with SLE correlate with cognitive impairment, depression and disease activity.

**WHAT IS ALREADY KNOWN ON THIS TOPIC**

**WHAT THIS STUDY ADDS**

- Synthetic MRI data revealed a marked reduction in grey matter volume in certain regions of the brain in both patients with non-NPSLE and NPSLE.
- Brain structural changes in patients with SLE were found to be associated with cognitive performance, depression levels and disease activity.

**HOW THIS STUDY MIGHT AFFECT RESEARCH, PRACTICE OR POLICY**

- Identifying and quantifying structural changes in the brain of patients with NPSLE could provide valuable insights into the pathophysiological mechanisms underlying cognitive impairment, as well as assist in its clinical diagnosis and treatment.

## Introduction

 SLE is a chronic autoimmune disorder that affects multiple organs and systems, leading to significant morbidity and diminished quality of life for patients who suffer from this chronic debilitating disorder.^1^ Among the various organ systems involved, the nervous system is frequently affected and can be severe. According to the 19 classification criteria established by the American College of Rheumatology (ACR), SLE with central and/or peripheral nervous system involvement is termed neuropsychiatric SLE (NPSLE).^2^ NPSLE represents one of the most severe complications of SLE and is the second leading cause of mortality in this patient population, following lupus nephritis.^3^ Consequently, early diagnosis and intervention are crucial for NPSLE, which has been challenging given its highly variable clinical manifestations. In addition, the pathogenesis of NPSLE remains unclear, and there is a lack of gold standards currently for its diagnosis. This highlights an urgent need for more work to find effective biomarkers to identify neuropsychiatric symptoms in patients with SLE.

Conventional brain MRI can be used to assess intracranial lesions, such as cerebral haemorrhage and infarction, which also helps to exclude other potential causes of neuropsychiatric symptoms such as brain tumours or infection in patients with SLE. The most prevalent findings on conventional brain MRI in patients with NPSLE are non-specific high signal areas in the white matter (WM) and brain atrophy; however, approximately 35–42% of patients with NPSLE show no detectable abnormalities on the conventional brain MRI images.^4 5^ To address this challenge, advanced neuroimaging techniques such as voxel and surface-based morphological structural MRI have been used to assess grey matter (GM) and cortical thickness in patients with SLE. For instance, the study by Jung *et al*^6^ showed reduced cortical thickness in patients with NPSLE but not in patients with non-NPSLE when compared with healthy controls (HCs). Another recent study also found extensive cortical atrophy in patients with NPSLE but not in patients with non-NPSLE relative to the HCs.^7^ Conversely, additional studies reported significantly lower grey matter volume (GMV) and white matter volume (WMV),^8^ as well as significantly thinner cortex in specific brain regions in patients with non-NPSLE when compared with the HCs.^9^ These findings suggest that brain structural changes may occur in patients with SLE and may be associated with neuropsychiatric symptoms.

Synthetic MRI (SyMRI) has advantages over both conventional brain MRI and morphological structural MRI. SyMRI captures multiple image sequences in a single scan, thus reducing scan time, and quantifies brain volume, GM, WM and myelin content (MyC) through advanced tissue segmentation.^10 11^ Importantly, SyMRI generates quantitative maps of longitudinal relaxation time (T1), transverse relaxation time (T2) and proton density (PD), offering comprehensive data for research. SyMRI has been extensively applied in the study of neurological disorders, including Alzheimer’s disease (AD),^12^ multiple sclerosis,^13 14^ brain tumours^15^ and the evaluation of brain development in children.^16^ It effectively assesses structural changes in brain tissue and the extent of disease-related damage. A study using SyMRI to assess neonatal brain imaging demonstrated that the T1 and T2-weighted images generated by SyMRI exhibited diagnostic accuracy comparable to that of conventionally acquired images.^17^ The quantitative parameters obtained by SyMRI provide more information for distinguishing patients with AD from normal controls.^12^ The increased T1 and T2 values of bilateral hippocampus may reflect the severity of AD. Differences in the spatial distribution of abnormal myelin volume fraction (MVF) distribution in SyMRI may help characterise WM in patients with multiple sclerosis.^14^ However, SyMRI has not been applied in assessing brain structure in patients with SLE, and the brain structural data in patients with NPSLE remain scarce.

Here, we performed a prospective study enrolling patients with SLE and age, gender and education-matched HCs. All study participants underwent SyMRI scans and neuropsychological testing. We assessed alterations of GM using SyMRI parameters including T1, T2 and PD values, which were correlated with neuropsychological testing data and clinical variables. We aimed to identify the potential neural correlates and neuroimaging biomarkers for cognitive functioning and neuropsychiatric symptoms in patients with SLE.

## Methods

### Participants

All participants provided written informed consent for the study. The study patients were prospectively recruited from the outpatient rheumatology clinics at The First Affiliated Hospital of Guangxi Medical University, PR China, from September 2021 to December 2023. Age, gender and education-matched HCs were recruited through patients’ referrals, clinical fliers, community health fairs, online and print newspaper advertisements and social media during the same study duration.

Patients who met the 2019 European League Against Rheumatism and ACR Classification Criteria for SLE^18^ and who had no contraindications for brain MRI were enrolled into the study. Exclusion criteria for all participants included: (1) severe physical illness; (2) history of psychiatric disorders (schizophrenia, bipolar disorder, etc); (3) central nervous system disorders (head trauma, brain tumours, AD, etc) or major central nervous system manifestations not SLE related; (4) pregnancy; (5) contraindications to MRI; and (6) poor MRI image quality (scanning artefacts, motion artefacts, etc).

Clinical demographic information for all participants including age, gender, education, body mass index, disease duration and SLE Disease Activity Index (SLEDAI) was assessed during the current visit. In addition, laboratory data including serum complement components (C3 and C4), erythrocyte sedimentation rate (ESR) and immunoglobulins (IgA, IgG, IgM) were also recorded. All participants completed neuropsychological testing, depression and anxiety assessments within 24 hours of the SyMRI, which included the Mini-Mental State Examination (MMSE),^19^ Montreal Cognitive Assessment (MoCA),^20^ Digit Span Test (DST), Self-Rating Anxiety Scale (SAS) and Self-Rating Depression Scale (SDS).

### Image acquisition and processing

SyMRI data were obtained using a 3T scanner (SIGNA Premier GE Healthcare, Wisconsin, USA). T1-weighted (T1w) images of each participant were acquired using a 1 mm isotropic sagittal 3D magnetisation prepared rapid gradient echo sequence. Quantitative SyMRI parameters were obtained using the imaging technique based on an axial 2D multiple-dynamic multiple-echo (MDME) sequence.^21^ The key parameters of the MDME sequence included the following: image resolution=2.0 mm×2.0 mm and slice thickness/gap=2/0 mm.

**Figure 1:**
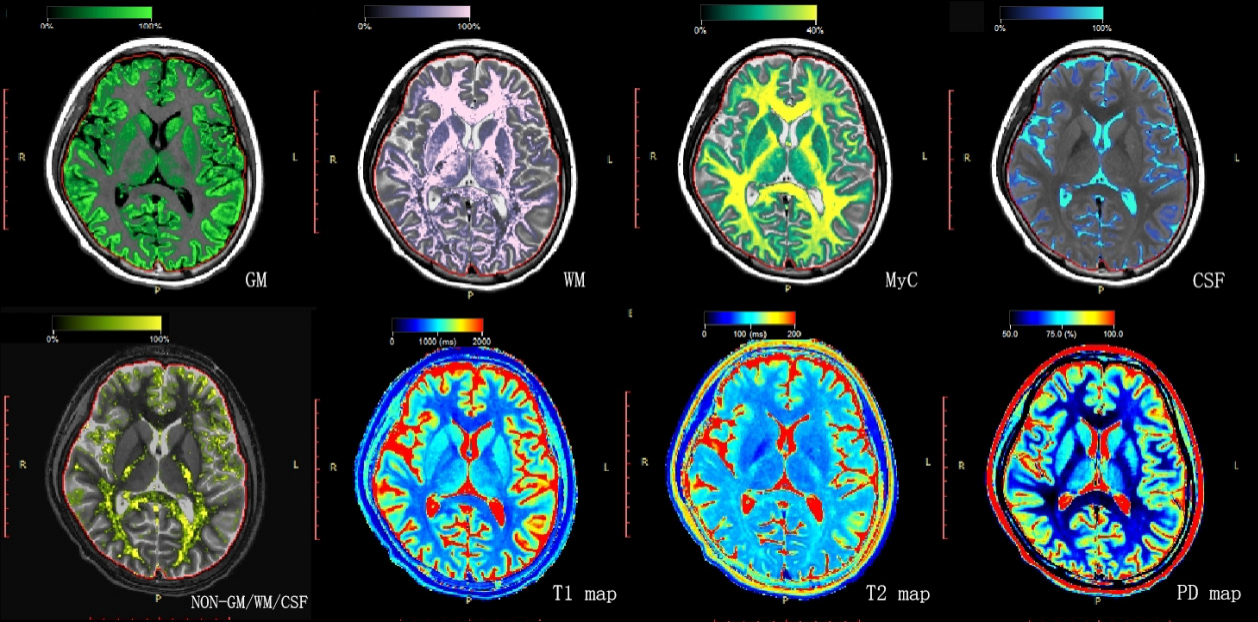
The grey matter volume, white matter volume, myelin content, cerebrospinal fluid volume and quantitative map of T1, T2 and PD were obtained by synthetic MRI (SyMRI). CSF, cerebrospinal fluid; GM, grey matter; MyC, myelin content; PD, proton density; T1, longitudinal relaxation time; T2, transverse relaxation time; WM, white matter.

SyMRI images were imported into SyMRI postprocessing software (V.11.2.2, SyntheticMR), and the GMV, WMV, brain parenchymal volume (BPV), intracranial volume (ICV), MyC, MVF, and mean T1, T2 and PD values of the GM were obtained (figure 1). The postprocessing software, SyntheticMR, was used to generate T1, T2 and PD volume content maps (T1m, T2m and PDm). For each participant, the process for extracting mean values of these quantitative maps in different brain regions was as follows: (1) bias fields of T1w images were removed using the Advanced Normalization Tools (ANTs); (2) the SynthStrip^22^ and SynthSeg^23^ algorithms embedded in the FreeSurfer software were used to generate brain masks and to segment the brain into three tissue types (GM, WM and cerebrospinal fluid); (3) the rigid transformation matrix between T1m and T1w images, as well as non-linear warped images between T1w images and T1w template images in Montreal Neurological Institute space, was computed using the ANTs-SyN algorithm; (4) the Automated Anatomical Labeling GM atlas^24^ was transformed into T1m space using the above transformation matrix and warped images; and (5) the GM and WM in the quantitative maps were segmented into specific brain regions using the transformed atlas, and the mean quantitative values (T1, T2 and PD) of each brain region were extracted.

### Statistical analysis

Data were analysed using SPSS V.22.0 and RStudio. The Shapiro-Wilk test was used to assess the normality of the data. The χ^2^ test assessed gender differences, while analysis of variance or the Kruskal-Wallis H test compared measures among three groups, followed by appropriate post hoc tests (t-test for parametric analyses and Mann-Whitney U test for non-parametric analyses). The Bonferroni method was used to correct for multiple comparisons. Pearson or Spearman correlations analysed relationships between imaging parameters and clinical data differing between the patients with SLE and the HCs. The differences in imaging parameters in brain regions were deemed significant at p<0.001 and with other analyses at p<0.05.

## Results

### Demographic and clinical data

The three groups, that is, the non-NPSLE, the NPSLE and the HC groups, were matched on age, gender and education. The SLEDAI values were increased in the NPSLE group as compared with patients with non-NPSLE (p=0.002), with no significant differences in disease duration or laboratory indices (p>0.05). Compared with the HC group, the non-NPSLE group had lower MoCA and DST scores and higher SAS and SDS scores, while the NPSLE group had lower MoCA, MMSE and DST scores (p<0.05). Table 1 presents the detailed clinical demographic information for all study participants. Online supplemental figure S1 shows the exclusion process of subjects.

**Table 1:** Clinical demographic data and neuropsychological assessment of patients with SLE and healthy controls (HCs)

	Non-NPSLE (n=57)	NPSLE (n=20)	HCs (n=29)	Kruskal-Wallis H test (P value)	Non-NPSLE versus NPSLE (P value)	Non-NPSLE versus HCs (P value)	NPSLE versus HCs (P value)	Non-NPSLE+NPSLE versus HCs (P value)
Gender (M/F)	6/51	2/18	7/22	0.194	–	–	–	0.070
Age (years)	28.44±7.7	24.65±6.67	29.76±10.77	0.115	–	–	–	0.534
BMI	21.12±3.38	20.68±3.32	21.22±3.01	0.645	–		–	0.728
Education (years)	12.6±2.79	11.75±2.31	12.83±3.27	0.213	–	–	–	0.349
MoCA	23.98±3.31	20.95±5.53	26.59±3.19	0.000*	0.088	0.001*	0.001*	0.000*
MMSE	27.14±1.82	25.4±3.57	28.03±1.86	0.006*	0.270	0.054	0.017*	0.005*
DST	13.05±1.97	11.7±2.47	14.86±2.71	0.000*	0.051	0.006*	0.000*	0.000*
SAS	44.88±11.93	42.2±9.73	37.48±6.12	0.015*	1.000	0.016*	0.183	0.005*
SDS	46.58±14.2	45.5±13.85	37.83±8.02	0.020*	1.000	0.019*	0.191	0.006*
Disease duration(months)	37.77±55.62	42.62±58.39	–	–	0.573	–	–	–
SLEDAI	10.96±5.91	16.7±7.18	–	–	0.002*	–	–	–
C3 (g/L)	0.58±0.32	0.48±0.2	–	–	0.409	–	–	–
C4 (g/L)	0.12±0.08	0.1±0.09	–	–	0.280	–	–	–
ESR (mm/hour)	35.32±26.01	34.4±27.52	–	–	0.732	–	–	–
IgG (g/L)	16.92±9.6	17.25±7.41	–	–	0.569	–	–	–
IgA (g/L)	2.45±1.34	2.16±0.98	–	–	0.504	–	–	–
IgM (g/L)	1.14±0.83	1.07±0.51	–	–	0.684	–	–	–

Data were presented as mean±SD.

* P<0.05.

BMI, body mass index; C3, C4, complement component; DST, Digit Span Test; ESR, erythrocyte sedimentation rate; IgA, IgG, IgM, immunoglobulin; MMSE, Mini-Mental State Examination; MoCA, Montreal Cognitive Assessment; Non-NPSLE, non-neuropsychiatric SLE; NPSLE, neuropsychiatric SLE; SAS, Self-Rating Anxiety Scale; SDS, Self-Rating Depression Scale; SLEDAI, SLE Disease Activity Index.

### SyMRI data

The brain segmentation data showed decreased values in GMV, BPV and brain parenchymal fraction (BPF) and an increased value in MVF in the non-NPSLE group as compared with the HC group (p<0.05). The GMV, WMV, BPV, BPF and MyC were all decreased in the NPSLE group (p<0.05). Compared with the non-NPSLE groups, the NPSLE group had decreased WMV, BPF and MyC (p<0.05). There were no statistically significant differences in ICV, GMV/BPV or WMV/BPV among the non-NPSLE, NPSLE and HC groups (all p>0.05). Table 2 presents the detailed brain segmentation results for all study participants.

**Table 2:** Comparison of synthetic MRI (SyMRI) automatic volume measurement and quantitative index results between patients with SLE and healthy controls (HCs)

	Non-NPSLE (n=57)	NPSLE (n=20)	HCs (n=29)	K-W test or ANOVA (P value)	Non-NPSLE versus NPSLE(P value)	Non-NPSLE versus HCs (P value)	NPSLE versus HCs (P value)	Non-NPSLE+NPSLE versus HCs (P value)
WMV (mL)	452.76±43.32	417.87±43.44	466.32±51.07	0.001*	0.004*	0.195	0.000*	0.030*
GMV (mL)	472.92±61.37	463.91±54.21	504.31±60.69	0.034*	0.564	0.024*	0.022*	0.011*
BPV (mL)	1106.16±98.76	1060.50±81.85	1179.55±102.71	0.000*	0.073	0.001*	0.000*	0.000*
ICV (mL）	1353.47±116.51	1341.20±86.06	1382.93±127.82	0.396	0.682	0.263	0.214	0.193
BPF (%)	81.77±3.17	79.09±3.92	85.36±2.54	0.000*	0.002*	0.000*	0.000*	0.000*
WMV/BPV	0.41±0.03	0.39±0.03	0.40±0.04	0.106	0.190	0.211	0.969	0.225
GMV/BPV	0.43±0.04	0.44±0.05	0.43±0.03	0.555	0.582	0.991	0.585	0.660
MyC (mL）	181.85±19.32	166.44±17.70	183.44±16.55	0.003*	0.002*	0.704	0.002*	0.182
MVF (%)	16.41±1.30	15.71±1.31	15.59±1.23	0.009*	0.036	0.006*	0.757	0.026*
WM T1 (ms)	851.04±14.97	865.65±20.92	846.83±14.46	0.000*	0.001*	0.255	0.000*	0.033
WM T2 (ms)	85.07±1.74	86.35±2.46	84.76±1.88	0.040*	0.058	1.000	0.066	0.320
WM PD (%)	66.87±0.93	67.23±1.05	67.41±0.94	0.041*	0.156	0.015*	0.514	0.036*
GM T1 (ms)	1462.53±42.45	1492.35±39.41	1416.10±29.99	0.000*	0.004*	0.000*	0.000*	0.000*
GM T2 (ms)	110.72±3.56	114.25±3.61	107.52±3.02	0.000*	0.003*	0.001*	0.000*	0.000*
GM PD (%)	82.74±0.83	83.17±0.89	83.35±0.84	0.005*	0.050	0.002*	0.470	0.009*

Data were presented as mean±SD.

* P<0.05.

ANOVA, analysis of variance; BPF, brain parenchymal fraction; BPV, brain parenchymal volume; GM, grey matter; GMV, grey matter volume; ICV, intracranial volume; K-W, Kruskal-Wallis; MVF, myelin volume fraction; MyC, myelin content; Non-NPSLE, non-neuropsychiatric SLE; NPSLE, neuropsychiatric SLE; PD, proton density; T1, longitudinal relaxation time; T2, transverse relaxation time; WM, white matter; WMV, white matter volume.

Regarding the T1 values, the NPSLE group had extensively higher GM T1 values than the HC group, including the 63 brain regions in the frontal, temporal, parietal, occipital, insula, limbic system and cerebellum (all p<0.001). The non-NPSLE group also had higher T1 values than the HC group in several brain regions, and some brain regions in the NPSLE group had higher T1 values than those in the non-NPSLE group (all p<0.001). Detailed data can be found in online supplemental table S1.

Regarding the T2 values, the NPSLE group had a wide range of T2 values higher than the HC group, including 80 brain regions in the frontal, temporal, parietal, occipital, insula, limbic system and cerebellum (all p<0.001). The non-NPSLE group had higher T2 values than the HC group in multiple brain regions, while the non-NPSLE group had multiple brain regions with lower T2 values than the NPSLE group (all p<0.001). Detailed data can be found in online supplemental table S2.

Regarding the PD values, the NPSLE group had reduced PD values in the right dorsolateral superior frontal gyrus and bilateral medial superior frontal gyrus, and increased PD values in the right lingual gyrus when compared with the HC group (p<0.001). There were reduced PD values in several brain regions in the non-NPSLE group when compared with the HC group (p<0.001). PD values were increased in the left crus II of the cerebellar hemisphere and left cerebellar 7b in the NPSLE group as compared with the non-NPSLE group (p<0.001). Detailed data can be found in online supplemental table S3.

### Correlation analysis data

The SyMRI parameters from the brain segmentation including the BPV, BPF, MVF, WM PD, GM T1 and GM PD were significantly correlated with cognitive functioning scores, SDS scores, SLEDAI and laboratory indicators (p<0.05). WMV and GMV were not significantly correlated with cognitive and neuropsychological assessment scales or clinical indicators (p>0.05) (table 3).

**Table 3:** Correlation analysis of synthetic MRI (SyMRI) automatic segmentation measurement with neuropsychological assessment scores and clinical indicators

	WMV	GMV	BPV	BPF	MVF	WM T1	WM PD	GM T1	GM T2	GM PD
MoCA	0.175	0.091	0.204	0.21	0.106	−0.101	−0.089	−0.234*	−0.132	−0.168
MMSE	0.129	−0.111	−0.018	0.129	0.239*	−0.152	−0.188	−0.241*	−0.167	−0.293†
DST	0.079	0.047	0.082	0.029	−0.011	−0.019	0.069	−0.033	−0.086	−0.005
SAS	0.056	0.114	0.134	−0.095	−0.174	0.137	0.157	0.183	0.128	0.192
SDS	0.059	0.216	0.188	−0.17	−0.151	0.145	0.141	0.303†	0.193	0.248*
SLEDAI	−0.097	−0.201	−0.231*	−0.246*	−0.005	0.208	0.12	0.319†	0.221	0.011
C3	−0.025	0.047	0.13	0.066	0.103	−0.118	−0.233*	−0.132	−0.01	−0.08
C4	−0.072	−0.049	0.087	0.095	0.168	−0.149	−0.303†	−0.107	−0.012	−0.193
ESR	0.061	−0.038	−0.051	0.047	0.011	0.016	0.075	0.126	0.046	0.171
IgG	0.193	0.013	−0.02	−0.015	0.027	0.009	0.169	0.113	−0.054	0.290*
IgA	0.102	0.084	0.02	0.132	−0.002	−0.017	0.164	−0.151	−0.222	0.029
IgM	0.01	0.03	0.012	0.088	−0.15	0.146	0.313†	−0.037	0.026	0.116

* P<0.05.

† P<0.01.

BPF, brain parenchymal fraction; BPV, brain parenchymal volume; C3, C4, complement component; DST, Digit Span Test; ESR, erythrocyte sedimentation rate; GM, grey matter; GMV, grey matter volume; IgA, IgG, IgM, immunoglobulin; MMSE, Mini-Mental State Examination; MoCA, Montreal Cognitive Assessment; MVF, myelin volume fraction; PD, proton density; SAS, Self-Rating Anxiety Scale; SDS, Self-Rating Depression Scale; SLEDAI, SLE Disease Activity Index; T1, longitudinal relaxation time; T2, transverse relaxation time; WM, white matter; WMV, white matter volume.

**Figure 2:**
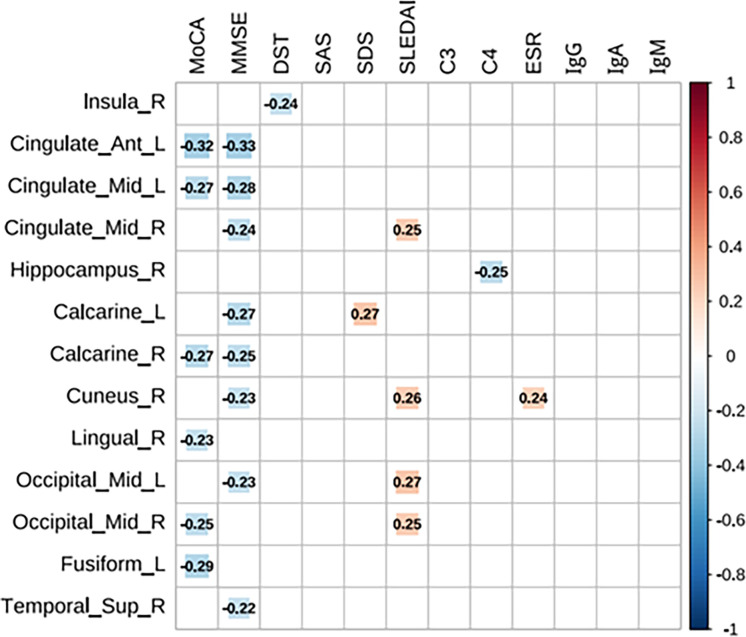
Brain regions with significant correlations between longitudinal relaxation time (T1) values and clinical data in patients with SLE. C3, C4, complement component; DST, Digit Span Test; ESR, erythrocyte sedimentation rate; Ig, immunoglobulin; MMSE, Mini-Mental State Examination; MoCA, Montreal Cognitive Assessment; SAS, Self-Rating Anxiety Scale; SDS, Self-Rating Depression Scale; SLEDAI, SLE Disease Activity Index.

The T1 values in the left anterior cingulate and paracingulate gyri, left median cingulate and paracingulate gyri, and right calcarine fissure and surrounding cortex in patients with SLE showed negative correlations with MoCA and MMSE scores. T1 values in the right insula were negatively correlated with DST. T1 values in the right hippocampus negatively correlated with C4 levels. Conversely, T1 values in the left calcarine fissure and surrounding cortex were positively correlated with SDS scores. Additionally, T1 values in the right median cingulate and paracingulate gyri, right cuneus and bilateral middle occipital gyrus were positively correlated with SLEDAI scores, while T1 values in the right cuneus were positively correlated with ESR. Detailed data can be found in figure 2.

The T2 values in the left median cingulate and paracingulate gyri were negatively correlated with MoCA and MMSE scores. T2 values in the left calcarine fissure and surrounding cortex, right inferior occipital gyrus, left fusiform gyrus and left superior temporal gyrus were positively correlated with SDS scores. Additionally, T2 values in the right inferior occipital gyrus were positively correlated with SLEDAI scores, while T2 values in the bilateral calcarine fissure and surrounding cortex were positively correlated with ESR. Detailed data can be found in online supplemental figure S2.

The PD values in the right superior frontal gyrus (medial) were positively correlated with the SDS scores and IgM levels and negatively correlated with the C3 and C4 levels (online supplemental figure S3).

## Discussion

In this study, SyMRI data revealed a significant reduction in the total GMV of the brain in both patients with non-NPSLE and NPSLE compared with HCs. In addition, brain structural alterations in patients with SLE were correlated with cognitive functioning, depression score and disease activity. To the best of our knowledge, this was the first prospective study using the SyMRI technique to quantitatively assess GM regional alteration and its potential association with cognitive functioning in patients with SLE.

The results of the study align with published literature on structural MRI, which reported extensive cortical and subcortical atrophy^7^ and reduced cortical thickness across multiple brain regions in patients with NPSLE.^25^ For patients with non-NPSLE, our study showing cortical atrophy was also similar to a prior report of cortical volume loss.^9^ These findings suggest that brain atrophy may occur in patients with SLE even before the onset of neuropsychiatric symptoms. Autoimmune responses in SLE may lead to disruption of the blood-brain barrier and neuronal damage, contributing to brain atrophy.^3^ Existing literature has indicated an association between glucocorticoid use and brain atrophy in patients with SLE.^8 26 27^ Further research is needed to elucidate the pathophysiology underlying brain atrophy in patients with SLE.

This study identified a significant reduction in the whole-brain myelin as measured by MyC in patients with NPSLE as compared with the HCs. NPSLE has been thought to be associated with autoimmune inflammatory processes, where autoantibodies targeting the central nervous system may induce inflammation and demyelination.^4^ The demyelination level in the NPSLE group may be decreased as indicated by the MyC values in this study.

We observed significantly increased T1 and T2 values across multiple brain regions including the frontal, temporal, parietal and occipital lobes, as well as the cerebellum in patients with SLE, with more pronounced changes in patients with NPSLE. Potential explanations for this finding are as follows. First, the T1 and T2 parameters are tissue-specific values that play a crucial role in differentiating various tissue types. Previous studies have demonstrated that alterations of T1 and T2 values correlate with factors such as age, tissue water content, iron deposition and myelin integrity.^28^,^32^ The brain is a primary target organ affected by SLE through a multifaceted interaction of inflammatory mediators, vascular endothelial cells and immune complexes that contributes to the pathophysiology of NPSLE.^3^ Patients with NPSLE frequently exhibit central nervous system inflammation, oedema and demyelination. Oedema contributes to an increased water content in brain tissue, leading to elevated T1 and T2 values. Second, myelin loss has been shown to elevate T1 values.^31^ Notably, global brain atrophy may compress the free water spaces, resulting in a decreased ratio of free water to restricted water, which in turn may lower T2 values.^33^ Based on these observations, we speculate that inflammation and oedema may be the dominant contributors to brain injury in patients with NPSLE. Third, iron is known to prolong the relaxation time of hydrogen atoms, resulting in increased T1 values. A prior study assessing quantitative susceptibility mapping values in the basal ganglia of patients with SLE found evidence of increased iron deposition, which correlates with disease progression.^34^ Thus, the elevated GM T1 values in patients with SLE may be associated with increased iron deposition in the brain, though further research is needed to confirm these interpretations. Lastly, patients with SLE have disruption of the blood-brain barrier and small vessel lesions that may lead to alterations in local blood flow and metabolism.^4^ These changes may significantly affect the T1 and T2 parameters.

Brain regions exhibiting significantly elevated T1 and T2 values were identified in patients with NPSLE when compared with patients with non-NPSLE, indicating that T1 and T2 values may reflect the extent of structural damage since patients with NPSLE were expected to have more severe brain injury than patients with non-NPSLE. Therefore, the T1 and T2 values may provide valuable insights for the early diagnosis and clinical management of NPSLE.

Our study revealed a more pronounced decrease in PD values among patients with non-NPSLE compared with those with NPSLE. The PD parameter reflects the density of hydrogen atoms in tissue and is typically positively correlated with water content.^35^ In patients with NPSLE, we speculate the extensive detrimental effects of inflammation, oedema and blood-brain barrier disruption obscuring local changes in water content and resulting in the lack of a notable decline in PD values. On the other hand, patients with non-NPSLE may have less damaging effects from neural inflammation and thus show a more distinct reduction in water content as compared with patients with NPSLE. This variation may reflect the heterogeneity of pathophysiological mechanisms present across different subsets of patients with SLE. Further research is needed to explore the significance of PD values in the quantitative assessment of brain structure in patients with SLE.

This study identified an association between brain atrophy as indicated by the SyMRI brain volume parameters and the SLE disease activity assessed with the SLEDAI scale. Previous research has also reported a correlation between localised reductions in brain volume and SLEDAI in patients with SLE.^36 37^ However, our study did not find a significant correlation between brain atrophy and neuropsychological assessment scales. It might be due to the SyMRI measuring the whole brain volume and therefore could not directly reflect the relationships between focal brain atrophy and cognitive changes which may occur in specific brain regions.

We observed that cortical T1 and/or T2 values were negatively correlated with scores on the MoCA and/or MMSE across multiple brain regions, including the left anterior cingulate gyrus, paracentral cingulate gyrus, bilateral medial and paracentral cingulate gyri, bilateral periaqueductal fissure cortex, right cuneate lobe, right lingual gyrus, bilateral middle occipital gyrus, left fusiform gyrus and right supratemporal gyrus. Furthermore, many of these regions exhibited positive correlations with SLEDAI and ESR, suggesting that structural damage in these areas is associated with cognitive impairment and disease activity. Additionally, the PD values in the bilateral medial superior frontal gyrus were negatively correlated with complement levels for C3 and/or C4, while PD values in the left dorsolateral superior frontal gyrus and bilateral medial superior frontal gyrus were positively correlated with IgM levels. The complement system, autoantibodies and inflammatory mediators play crucial roles in the complex pathophysiological processes of SLE.^3^ We therefore speculate that C3, C4 and IgM may contribute to brain structural damage in patients with SLE. The structural and functional integrity of the cerebral cortex is essential for the normal execution of cognitive functions. Several studies have established a link between structural abnormalities in the cerebral cortex and cognitive dysfunction in patients with SLE. For instance, Zimmermann *et al*^38^ reported significantly smaller volumes of the left hippocampus, amygdala and right hippocampus in patients with SLE and cognitive impairment compared with those with normal cognitive function. Additionally, Bizzo *et al*^25^ found reduced cortical thickness in the left supramarginal and superior temporal gyri in patients with SLE and situational memory deficits, as well as abnormalities in the right superior frontal gyrus, caudal gyrus, rostral middle frontal gyrus and precentral gyrus compared with patients with SLE without such deficits. Our study identified overlapping brain regions as reported in the literature, which may potentially be the neural correlates for cognitive functioning in patients with SLE.

Patients with SLE may experience anxiety and depression.^39 40^ A study employing resting-state functional MRI found abnormalities in brain function being associated with anxiety or depression in patients with SLE.^41^ Our study also showed that patients with SLE had higher SAS and SDS scores for anxiety and depression than the HCs. In addition, the T1 and T2 values of the left perisylvian fissure cortex; the T2 values of the right inferior occipital gyrus, left fusiform gyrus and left superior temporal gyrus; and the PD values of the right medial superior frontal gyrus were positively correlated with SDS scores. These brain regions are critical for the generation and response of emotions, suggesting that abnormalities in these cortical structures may underlie the depressive symptoms observed in patients with SLE. However, we did not identify any brain regions significantly correlated with SAS scores, suggesting potential differences in the neural pathways underlying anxiety and depression in this patient population.

There were limitations to this study. This study was limited by a small sample size, especially the patients with NPSLE. In addition, a cross-sectional study design rendered a lack of follow-up data for the patients in this cohort. Future prospective longitudinal studies using SyMRI are needed to investigate the long-term changes in brain structure and their correlations with cognitive function in patients with SLE.

In summary, this study observed significant GM alterations in patients with SLE, which were correlated with their cognitive function, psychological well-being and disease activity. GM alterations may play a role in the pathogenesis of SLE, especially in patients with neuropsychiatric symptoms. Furthermore, SyMRI parameters such as T1, T2 and PD identified alterations in specific brain regions which may potentially serve as the neural correlates for cognitive functioning in patients with SLE.

## Supplementary material

10.1136/lupus-2025-001505 online supplemental file 1

## Data Availability

Data are available upon reasonable request.
