# Supplementary material for: Assessing grey matter structural alterations in systemic lupus erythematosus using synthetic MRI
Source: Lupus Sci Med. 2025 Jul 13;12(2):e001505. doi: 10.1136/lupus-2025-001505 (PMC12273088; doi:10.1136/lupus-2025-001505)
Supplement: online supplemental file 1 [file lupus-12-2-s001.pdf]

Supplementary files:

Table S1. Brain regions with different longitudinal relaxation time (T1) values (ms) between patients with systemic lupus erythematosus (SLE) and healthy controls (HCs)

	non-NPSLE（n=57）	NPSLE（n=20）	HCs（n=29）	Kruskal-Wallis H or ANOVA	non-NPSLE VS. NPSLE (*p*)	non-NPSLE VS. HCs（*p*）	NPSLE VS. HCs（*p*）	non-NPSLE + NPSLE VS. HCs（*p*）
Precentral_L	1176.35±50.06	1213.46±40.19	1164.99±40.15	0.000	0.001	1	0.000*	1
Frontal_Sup_R	1259.9±45.78	1284.2±34.36	1242.66±35.98	0.001	0.081	0.107	0.001*	0.494
Frontal_Inf_Oper_R	1218.66±42.35	1256.55±44.55	1199.96±35.73	0.000	0.002	0.146	0.000*	0.146
Frontal_Inf_Tri_L	1237.26±40.88	1265.28±37.96	1220.77±31.04	0.001	0.016	0.178	0.000*	0.264
Frontal_Inf_Tri_R	1244.9±43.64	1273.3±44.71	1219.21±29.32	0.000	0.072	0.019	0.000*	0.046
Frontal_Inf_Orb_L	1237.59±46.42	1274.5±48.37	1210.52±31.91	0.000	0.004	0.022	0.000*	0.003
Frontal_Inf_Orb_R	1253.37±45.89	1294.37±46.72	1236.75±29.19	0.000	0.001*	0.262	0.000*	0.090
Frontal_Med_Orb_L	1313.22±57.96	1347.11±56.33	1273.71±32.42	0.000	0.051	0.002	0.000*	0.004
Frontal_Med_Orb_R	1251±48.04	1286.76±57.77	1215.85±35.12	0.000	0.013	0.004	0.000*	0.000*
Rectus_L	1272.84±49.75	1306.69±48.82	1252.9±33.61	0.000	0.020	0.122	0.000*	0.412
Rectus_R	1230.77±47.93	1265.29±58.51	1203.85±37	0.000	0.018	0.043	0.000*	0.023
OFCant_R	1281.19±63.37	1312.67±59.41	1244.25±39.59	0.000	0.109	0.017	0.000*	0.004

Insula_R	1297.32±38.59	1327.12±35.87	1270.17±27.12	0.000	0.005	0.003	0.000*	0.000*
Cingulate_Ant_L	1318.57±37.3	1350.49±36.64	1286.82±32.7	0.000	0.005	0.000*	0.000*	0.000*
Cingulate_Ant_R	1244.19±34.6	1275.18±49.27	1224.89±28.67	0.000	0.004	0.066	0.000*	0.029
Cingulate_Mid_L	1261.85±41.37	1309.91±45.25	1214.37±38.08	0.000	0.000*	0.000*	0.000*	0.000*
Cingulate_Mid_R	1215.6±38.05	1254.58±44.78	1186.8±30.3	0.000	0.000*	0.003	0.000*	0.000*
Cingulate_Post_L	1187.71±53.74	1243.54±63.98	1172.97±56.57	0.000	0.001*	0.766	0.000*	1
Cingulate_Post_R	1181.82±69.36	1242.45±70.17	1168.14±46.06	0.001	0.005	1	0.000*	1
Hippocampus_L	1309.97±44.19	1327.07±48.42	1264.01±34.19	0.000	0.376	0.000*	0.000*	0.000*
Hippocampus_R	1313.31±49.25	1351.57±52.73	1275.57±39.58	0.000	0.008	0.002	0.000*	0.000*
Calcarine_L	1265.91±57.12	1313.37±49.15	1213.65±46.08	0.000	0.002	0.000*	0.000*	0.000*
Calcarine_R	1254.9±61.48	1299.48±62.24	1196.68±44.45	0.000	0.011	0.000*	0.000*	0.000*
Cuneus_L	1253.88±58.65	1305.61±55.92	1211.49±50.85	0.000	0.002	0.004	0.000*	0.002
Cuneus_R	1240.12±64.54	1299.79±64.07	1189.55±53.05	0.000	0.001*	0.001	0.000*	0.000*
Lingual_L	1231.42±58.63	1268.32±53.47	1189.41±44.35	0.000	0.030	0.003	0.000*	0.001*
Lingual_R	1221.25±62.97	1278.29±42.56	1175.81±43.85	0.000	0.000	0.001	0.000*	0.000*
Occipital_Sup_L	1260.36±65.45	1321.73±48.36	1227.46±48.3	0.000	0.000*	0.045	0.000*	0.011
Occipital_Sup_R	1231.26±64.69	1283.45±56.87	1184.5±50	0.000	0.003	0.003	0.000*	0.000*
Occipital_Mid_L	1208.41±54.37	1254.55±39.95	1169.23±43.59	0.000	0.001	0.002	0.000*	0.001*
Occipital_Mid_R	1239.17±56.79	1279.83±55.54	1192.59±41.38	0.000	0.011	0.001*	0.000*	0.000*
Occipital_Inf_L	1208.47±57.37	1252.68±44.18	1176.85±46.01	0.000	0.005	0.027	0.000*	0.020
Occipital_Inf_R	1229.47±58.06	1276.01±54.33	1200.18±47.31	0.000	0.004	0.062	0.000*	0.052

Fusiform_L	1276.49±40.46	1304.38±32.32	1243.63±32.07	0.000	0.013	0.001*	0.000*	0.000*
Fusiform_R	1236.93±38.23	1268.77±33.27	1219.41±38	0.000	0.004	0.126	0.000*	0.389
Postcentral_L	1194.26±47.66	1222.84±41.27	1167.63±40	0.000	0.046	0.030	0.000*	0.055
Postcentral_R	1223.63±51.71	1242.81±37.86	1193.25±40.95	0.000	0.159	0.008	0.000*	0.027
Parietal_Sup_R	1262.51±54.99	1288.36±45.62	1230.48±41	0.000	0.146	0.017	0.000*	0.022
Parietal_Inf_L	1224.68±50.34	1260.85±44.34	1197.37±47.61	0.000	0.003	0.049	0.000*	0.149
Parietal_Inf_R	1243.5±50.64	1262.47±52.38	1204.12±38.08	0.000	0.380	0.001*	0.000*	0.003
Angular_L	1205.02±50.63	1238.65±41.13	1190.26±38.73	0.001	0.015	0.273	0.001*	1
Angular_R	1249.59±48.68	1274.9±50.07	1213.08±37.71	0.000	0.113	0.002	0.000*	0.001
Precuneus_L	1213.84±44.15	1248.9±36.6	1195.2±43.64	0.000	0.006	0.175	0.000*	0.656
Precuneus_R	1196.58±45.76	1242.1±38.9	1175.4±39.42	0.000	0.000*	0.098	0.000*	0.075
Putamen_R	1002.86±27.8	1020.27±28.77	989.41±26.03	0.001	0.050	0.103	0.001*	0.390
Heschl_R	1169.95±113.48	1222.66±80.83	1126.01±62.23	0.000	0.024	0.169	0.000*	0.451
Temporal_Sup_L	1230.17±44.5	1274.17±48.73	1199.83±38.61	0.000	0.001*	0.009	0.000*	0.003
Temporal_Sup_R	1229.22±36.7	1257.44±41.35	1202.51±28.89	0.000	0.009	0.004	0.000*	0.001*
Temporal_Mid_L	1245.47±36.15	1285.95±46.32	1222.96±28.98	0.000	0.000*	0.024	0.000*	0.002
Temporal_Mid_R	1266.82±29.74	1304.22±42.01	1238.97±28.07	0.000	0.000*	0.001*	0.000*	0.000*
Temporal_Inf_L	1263.47±36.56	1291.23±36.15	1246.18±35.1	0.000	0.011	0.114	0.000*	0.339
Temporal_Inf_R	1281.46±34.48	1312.82±27.86	1267.23±29.41	0.000	0.001*	0.162	0.000*	0.199
Cerebelum_Crus1_L	1366.47±59.41	1397.64±73.45	1323.71±51.74	0.000	0.149	0.007	0.000*	0.010
Cerebelum_Crus1_R	1350.86±58.12	1390.71±92.28	1307.18±50.22	0.000	0.055	0.010	0.000*	0.005
Cerebelum_Crus2_L	1345.9±66.25	1386.12±72.49	1307.79±56.84	0.000	0.058	0.035	0.000*	0.060

Cerebelum_Crus2_R	1366.72±67.27	1406.57±70.45	1322.63±60.58	0.000	0.067	0.013	0.000*	0.026
Cerebelum_4_5_L	1374.18±66.17	1400.42±74.52	1319.33±55.45	0.000	0.372	0.001	0.000*	0.001
Cerebelum_4_5_R	1371.55±63.76	1388.39±92.6	1300.3±53.2	0.000	1	0.000*	0.001*	0.000*
Cerebelum_6_L	1289.57±57.17	1318.48±66.79	1241.1±46.86	0.000	0.156	0.001*	0.000*	0.000*
Cerebelum_6_R	1299.77±58.3	1330.14±73.7	1244.63±47.04	0.000	0.148	0.000*	0.000*	0.000*
Cerebelum_7b_R	1327.64±72.02	1370.87±65.34	1300.05±58.33	0.000	0.025	0.105	0.000*	0.265
Vermis_6	1414.08±77.25	1434.4±87.24	1342.9±70.28	0.000	0.944	0.000*	0.000*	0.001
Vermis_7	1329.15±72.55	1361.66±82.68	1275.35±76.03	0.000	0.380	0.002	0.001*	0.010

Data were presented by mean ± standard deviation. *$p < 0.001$; non-NPSLE, non-neuropsychiatric SLE; NPSLE, neuropsychiatric SLE; R, right; L, left; Sup, superior; Inf, Inferior; Med, medial; Mid, middle.

Table S2. Brain regions with different transverse relaxation time (T2) values (ms) between patients with systemic lupus erythematosus (SLE) and healthy controls (HCs)

	non-NPSLE（n=57）	NPSLE（n=20）	HCs（n=29）	Kruskal-Wallis H or ANOVA	non-NPSLE VS. NPSLE (*p*)	non-NPSLE VS. HCs（*p*）	NPSLE VS. HCs（*p*）	non-NPSLE+NPSLE VS. HCs（*p*）
Precentral_L	96.99±3.85	100.96±3.82	95.73±4.38	0.000	0.001*	0.511	0.000*	1
Precentral_R	96.52±4.81	99.13±3.26	94.7±3.81	0.000	0.004	0.330	0.000*	0.966
Frontal_Sup_R	105.89±4.58	108.66±3.51	104.21±3.76	0.001	0.008	0.673	0.001*	1
Frontal_Mid_L	106.22±4.3	109.77±4.15	104.53±3.97	0.000	0.001	0.593	0.000*	1
Frontal_Mid_R	105.95±4.47	108.72±3.8	103.99±3.37	0.000	0.006	0.093	0.000*	0.284
Frontal_Inf_Oper_L	101.76±3.35	104.49±3.52	100.15±3.52	0.000	0.008	0.126	0.000*	0.480
Frontal_Inf_Oper_R	99.85±3.58	103.18±3.93	98.33±3.32	0.000	0.002	0.199	0.000*	0.327
Frontal_Inf_Tri_L	104.97±3.72	109.15±5.23	103.32±3.36	0.000	0.002	0.203	0.000*	0.603
Frontal_Inf_Tri_R	102.5±4.17	105.08±3.74	100.47±3.57	0.001	0.039	0.079	0.000*	0.196
Frontal_Inf_Orb_L	105.46±4.45	110.53±6.22	103.09±3.88	0.000	0.003	0.114	0.000*	0.199
Frontal_Inf_Orb_R	101.83±4.17	106.41±3.82	99.81±3.59	0.000	0.000*	0.083	0.000*	0.039
Rolandic_Oper_L	100.44±3.92	103.91±5.22	97.71±3.07	0.000	0.004	0.010	0.000*	0.001
Rolandic_Oper_R	99.74±4.64	102.28±4.44	97.13±3.58	0.000	0.080	0.029	0.000*	0.034
Olfactory_L	105.32±4.71	107.68±4.61	102.87±3.35	0.001	0.120	0.046	0.001*	0.048

Frontal_Med_Orb_L	110.12±5.02	114±6.23	108.08±3.89	0.001	0.029	0.183	0.000*	0.687
Frontal_Med_Orb_R	105.87±4.18	109.96±4.79	104.02±3.73	0.000	0.005	0.375	0.000*	1
Rectus_L	106.09±4.13	109.96±4.84	104.67±3.81	0.000	0.003	0.381	0.000*	1
Rectus_R	102.84±4.09	107.35±5.34	101.07±4.05	0.000	0.002	0.234	0.000*	0.782
OFCmed_L	104.26±4.12	107.54±5.12	102.68±3.19	0.002	0.048	0.300	0.001*	1
OFCmed_R	102.02±4.4	105.94±5.53	100.89±4.33	0.001	0.004	0.849	0.001*	1
OFCant_L	105.88±4.27	109.64±4.92	104.13±4.06	0.000	0.004	0.242	0.000*	0.556
OFCant_R	104.25±4.67	107.31±4.72	101.79±3.68	0.000	0.028	0.050	0.000*	0.051
OFCpost_L	102.2±4.35	106±4.57	101.19±3.75	0.000	0.002	0.903	0.001*	1
Insula_L	102.7±3	105.6±3.7	101.31±2.59	0.000	0.001	0.146	0.000*	0.116
Insula_R	105.7±3.52	109.1±3.61	103.21±2.74	0.000	0.001*	0.004	0.000*	0.000*
Cingulate_Ant_L	107.79±3.52	111.04±3.48	105.7±3.27	0.000	0.001	0.046	0.000*	0.055
Cingulate_Ant_R	103.18±2.94	106.2±4.13	101.99±3.11	0.001	0.014	0.172	0.000*	0.687
Cingulate_Mid_L	101.62±3.76	106.29±4.05	98.06±3.57	0.000	0.000*	0.000	0.000*	0.000*
Cingulate_Mid_R	97.71±3.32	101.23±3.48	95.45±3.07	0.000	0.000*	0.009	0.000*	0.004
Cingulate_Post_L	91.57±3.8	96.5±4.64	89.46±3.76	0.000	0.000*	0.064	0.000*	0.089
Cingulate_Post_R	89.32±4.26	94.87±5.37	87.94±2.92	0.000	0.000*	0.512	0.000*	1
Hippocampus_L	109.27±4.08	113.03±4.01	105.47±2.59	0.000	0.001*	0.000*	0.000*	0.000*
Hippocampus_R	109.77±4.15	114.66±5.33	106.21±3.27	0.000	0.001	0.001*	0.000*	0.001*
ParaHippocampal_L	102.41±3.56	105.26±2.9	100.1±2.97	0.000	0.004	0.008	0.000*	0.005
ParaHippocampal_R	100.8±2.97	103.71±2.91	98.96±2.86	0.000	0.001*	0.021	0.000*	0.021

Calcarine_L	99.32±5.82	104.22±6.22	93.48±4.86	0.000	0.004	0.000*	0.000*	0.000*
Calcarine_R	98.86±5.91	103.22±7.12	92.65±4.39	0.000	0.056	0.000*	0.000*	0.000*
Cuneus_L	97.47±5.3	103.26±5.75	93.5±4.94	0.000	0.000*	0.004	0.000*	0.001
Cuneus_R	95.84±5.31	101.86±6.94	91.74±5.59	0.000	0.003	0.002	0.000*	0.003
Lingual_L	95.4±4.79	98.82±4.33	91.12±4.21	0.000	0.008	0.000*	0.000*	0.000*
Lingual_R	93.69±4.58	98.25±4.33	89.34±3.88	0.000	0.000*	0.000*	0.000*	0.000*
Occipital_Sup_L	97.5±5.74	103.74±5.35	93.71±4.64	0.000	0.000*	0.008	0.000*	0.001*
Occipital_Sup_R	94.39±5.1	100.63±6.55	90.6±4.7	0.000	0.001	0.005	0.000*	0.006
Occipital_Mid_L	93.81±5.27	97.78±3.83	90.64±4.02	0.000	0.001*	0.012	0.000*	0.011
Occipital_Mid_R	94.98±4.5	99.66±5.2	91.77±3.8	0.000	0.001*	0.004	0.000*	0.005
Occipital_Inf_L	92.88±4.39	97.58±3.85	90.29±3.95	0.000	0.000*	0.023	0.000*	0.011
Occipital_Inf_R	92.29±4.14	97.85±4.89	88.71±3.64	0.000	0.000*	0.002	0.000*	0.001*
Fusiform_L	99.51±3.65	102.92±3.36	96.88±2.9	0.000	0.001*	0.003	0.000*	0.000*
Fusiform_R	96.6±3.27	99.67±3.45	94.54±3.25	0.000	0.002	0.022	0.000*	0.028
Postcentral_L	98.28±4.12	101.52±3.92	95.47±3.98	0.000	0.004	0.013	0.000*	0.020
Postcentral_R	99.87±4.7	102.56±3.87	97.15±3.88	0.000	0.016	0.044	0.000*	0.104
Parietal_Sup_R	101.28±6.06	104.78±4.92	98.39±5.01	0.000	0.008	0.145	0.000*	0.451
Parietal_Inf_L	98.06±4.12	101.45±4.04	95.59±4.41	0.000	0.007	0.033	0.000*	0.116
Parietal_Inf_R	100.23±4.67	103.03±3.71	97.54±4.23	0.000	0.018	0.100	0.000*	0.259
SupraMarginal_R	100.7±3.71	102.99±2.74	98.78±3.3	0.000	0.006	0.136	0.000*	0.327
Angular_L	95.68±3.85	98.94±4.07	94.4±3.74	0.000	0.005	0.447	0.000*	1

Angular_R	98.44±3.94	101.73±3.63	96.14±3.97	0.000	0.005	0.033	0.000*	0.083
Precuneus_L	97.39±4.35	100.86±3.52	95.51±4.04	0.000	0.002	0.187	0.000*	0.412
Precuneus_R	95.05±4.95	98.41±3.56	92.8±3.94	0.000	0.001*	0.158	0.000*	0.284
Heschl_L	101.09±6.86	105.06±5.77	97.74±5.42	0.001	0.051	0.065	0.000*	0.117
Heschl_R	97.29±9.12	101.98±8.12	92.52±4.6	0.000	0.021	0.043	0.000*	0.131
Temporal_Sup_L	100.59±4.03	104.87±4.58	97.58±3.52	0.000	0.000*	0.004	0.000*	0.001*
Temporal_Sup_R	100.24±3.99	102.97±3.74	97.41±3.31	0.000	0.019	0.004	0.000*	0.003
Temporal_Pole_Sup_L	117.91±5.54	122.14±4.19	116.57±4.64	0.000	0.002	0.972	0.000*	1
Temporal_Mid_L	98.63±3.56	103.1±3.8	96.76±3.01	0.000	0.000*	0.089	0.000*	0.077
Temporal_Mid_R	100.54±3.47	104.48±4.47	97.95±3.17	0.000	0.002	0.008	0.000*	0.011
Temporal_Inf_L	99.58±3.22	103.47±3.46	98.24±3.11	0.000	0.000*	0.218	0.000*	0.202
Temporal_Inf_R	100.39±3.68	103.71±3.5	98.57±2.96	0.000	0.001	0.070	0.000*	0.039
Cerebelum_Crus1_L	109.6±4.7	112.23±3.89	106.23±4.72	0.000	0.036	0.002	0.000*	0.007
Cerebelum_Crus1_R	106.86±4.6	109.44±5.89	103.22±4.3	0.000	0.139	0.000*	0.000*	0.000*
Cerebelum_Crus2_L	105.67±4.5	109.14±4.54	102.95±4.17	0.000	0.014	0.028	0.000*	0.057
Cerebelum_Crus2_R	104.63±4.08	107.45±4.2	101.02±3.71	0.000	0.018	0.000*	0.000*	0.000*
Cerebelum_4_5_L	112.01±5.79	114.93±5.33	107.53±4.51	0.000	0.061	0.001	0.000*	0.003
Cerebelum_4_5_R	110.47±6.05	112.75±6.78	104.75±4.09	0.000	0.550	0.000*	0.000*	0.000*
Cerebelum_6_L	104.51±4.36	107.35±3.88	101.7±3.74	0.000	0.018	0.005	0.000*	0.008
Cerebelum_6_R	105.48±4.91	107.71±4.8	101.5±3.92	0.000	0.151	0.000*	0.000*	0.000*

Cerebelum_7b_R	100.7±5.08	102.68±3.64	97.4±2.92	0.000	0.049	0.005	0.000*	0.008
Cerebelum_8_L	100.26±3.69	102.71±6.06	97.63±2.83	0.000	0.081	0.008	0.000*	0.020
Cerebelum_8_R	97.49±3.85	99.16±3.69	94.96±2.74	0.000	0.155	0.022	0.000*	0.066
Cerebelum_9_L	100.47±3.11	102.46±4.29	99.01±2.4	0.002	0.202	0.085	0.000*	0.351

Data were presented by mean ± standard deviation. *$p < 0.001$; non-NPSLE, non-neuropsychiatric SLE; NPSLE, neuropsychiatric SLE; R, right; L, left; Sup, superior; Inf, Inferior; Med, medial; Mid, middle.

Table S3. Brain regions with different proton density (PD) values (ms) between patients with systemic lupus erythematosus (SLE) and healthy controls (HCs)

	non-NPSLE （n=57）	NPSLE （n=20）	HCs （n=29）	ANOVA	non-NPSLE VS. NPSLE (p)	non-NPSLE VS. HCs （p）	NPSLE VS. HCs （p）	non-NPSLE+ NPSLE VS. HCs （p）
Frontal_Sup__L	82.1±1.75	82.45±1.63	84.34±2.03	0.000	1	0.000*	0.001	0.001*
Frontal_Mid__L	82.47±1.86	82.86±2.07	84.57±2.41	0.000	1	0.000*	0.016	0.028
Frontal_Sup_Medial_L	80.28±1.54	80.7±1.57	82.53±1.83	0.000	0.976	0.000*	0.001	0.000*
Frontal_Sup_Medial_R	81.52±1.78	81.58±1.75	84.06±2.29	0.000	1	0.000*	0.000	0.000*
Frontal_Med_Orb_L	81.56±1.87	82.26±1.86	83.46±2.21	0.000	0.513	0.000*	0.115	0.067
Frontal_Med_Orb_R	81.6±1.79	82.38±1.75	83.59±2.66	0.000	0.433	0.000*	0.137	0.220
Rectus_L	80.65±1.51	81.12±1.43	82.14±1.5	0.000	0.675	0.000*	0.066	0.014
Cingulate_Ant_L	81.11±1.39	81.37±1.3	82.53±1.46	0.000	1	0.000*	0.015	0.009
Cingulate_Ant_R	81.1±1.36	81.51±1.37	82.49±1.79	0.000	0.853	0.000*	0.082	0.140
Lingual_R	78.41±1.05	78.99±0.88	77.91±0.79	0.001	0.067	0.075	0.001	0.129
Temporal_Pole_Sup_L	81.67±1.9	82.65±2.33	83.51±1.99	0.000	0.193	0.000*	0.433	0.079
Temporal_Mid_L	81.09±1.44	82.4±1.87	82.69±1.96	0.000	0.010	0.000*	1	0.433
Temporal_Pole_Mid_R	83.92±1.74	84.08±2.13	85.35±1.17	0.001	1	0.001*	0.032	0.002
Temporal_Inf_L	82.24±1.68	83.17±1.9	83.68±1.46	0.001	0.102	0.001*	0.888	0.092
Cerebelum_Crus2_L	84.16±1.75	86.08±1.72	85.02±1.94	0.000	0.000*	0.113	0.140	1
Cerebelum_7b_L	81.28±1.65	83.09±2.12	82.54±2	0.000	0.001*	0.011	0.900	1

Data were presented by mean ± standard deviation. *$p<0.001$; non-NPSLE, non-neuropsychiatric SLE; NPSLE, neuropsychiatric SLE; R, right; L, left; Sup, superior; Inf, Inferior; Med, medial; Mid, middle.

Figure S1. Flowchart of the excluded subjects

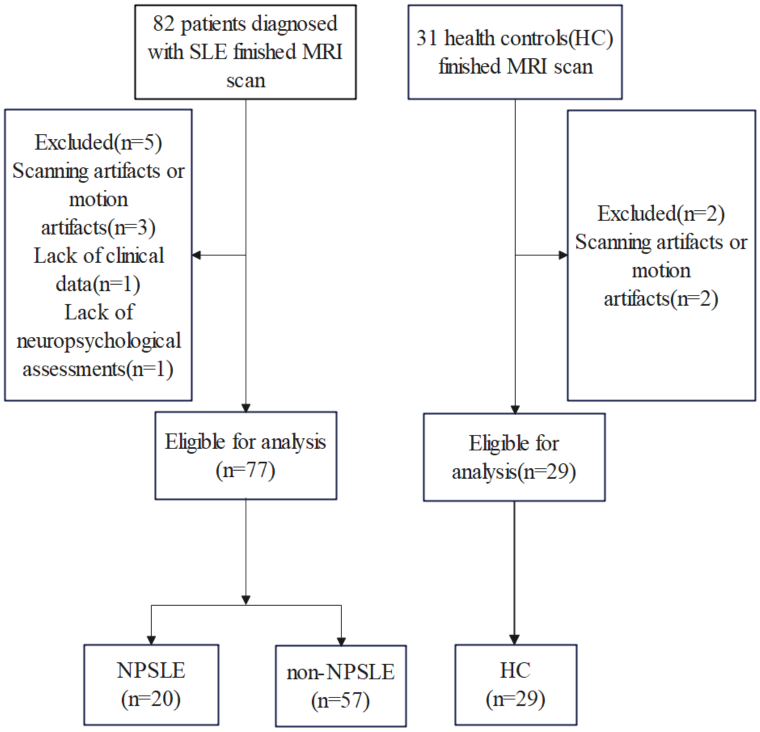

Figure S2. Brain regions with significant correlations between transverse relaxation time (T2) values and clinical data in patients with systemic lupus erythematosus (SLE)

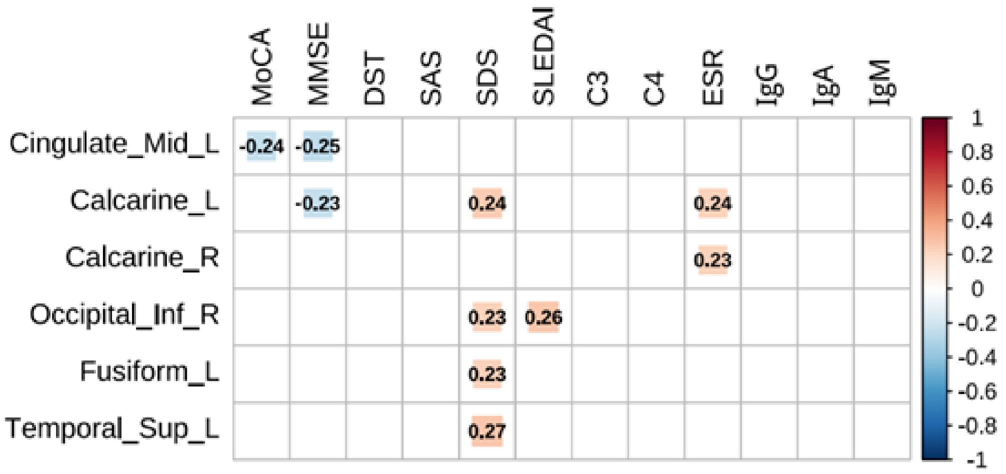

Abbreviations: R, right; L, left; Mid, middle; Inf, Inferior; Sup, superior; MoCA, Montreal Cognitive Assessment; MMSE, Mini-Mental State Examination; DST, Digit Sweepstakes Test; SAS, Self-assessed Anxiety Scale; SDS, Self-assessed Depression Scale; SLEDAI, Systemic Lupus Erythematosus Disease Activity Index; C3, C4, complement component; ESR, erythrocyte sedimentation rate; IgA, IgG, IgM, immunoglobulin.

Figure S3. Brain regions with significant correlation between proton density (PD) values and clinical data in in patients with systemic lupus erythematosus (SLE)

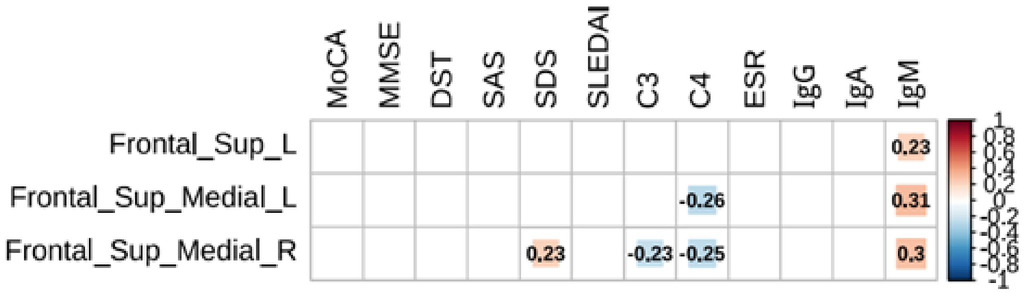

Abbreviations: R, right; L, left; Sup, superior; MoCA, Montreal Cognitive Assessment; MMSE， Mini-Mental State Examination; DST, Digit Sweepstakes Test; SAS, Self-assessed Anxiety Scale; SDS, Self-assessed Depression Scale; SLEDAI, Systemic Lupus Erythematosus Disease Activity Index; C3, C4, complement component; ESR, erythrocyte sedimentation rate; IgA, IgG, IgM, immunoglobulin.
